# Genome-wide analysis of AP2/ERF superfamily in lotus (*Nelumbo nucifera*) and the association between *NnADAP* and rhizome morphology

**DOI:** 10.1186/s12864-021-07473-w

**Published:** 2021-03-09

**Authors:** Dingding Cao, Zhongyuan Lin, Longyu Huang, Rebecca Njeri Damaris, Pingfang Yang

**Affiliations:** 1 Institute of Oceanography, Minjiang University, Fuzhou, 350108 China; 2 Institute of Cotton Research of the Chinese Academy of Agricultural Sciences, Anyang, China; 3 State Key Laboratory of Biocatalysis and Enzyme Engineering, School of Life Sciences, Hubei University, Wuhan, 430062 China

**Keywords:** *Nelumbo nucifera*, *AP2/ERF*, *NnADAP*, SNP, Rhizome

## Abstract

**Background:**

The AP2/ERF family is widely present in plants and plays a crucial regulatory role in plant growth and development. As an essential aquatic horticultural model plant, lotus has an increasingly prominent economic and research value.

**Results:**

We have identified and analysed the AP2/ERF gene family in the lotus. Initially, 121 AP2/ERF family genes were identified. By analysing their gene distribution and protein structure, and their expression patterns during the development of lotus rhizome, combined with previous studies, we obtained an SNP (megascaffold_20:3578539) associated with lotus rhizome phenotype. This SNP was in the *NnADAP* gene of the AP2 subfamily, and the changes in SNP (C/T) caused amino acid conversion (proline/leucine). We constructed a population of 95 lotus varieties for SNP verification. Through population typing experiments, we found that the group with SNP CC had significantly larger lotus rhizome and higher soluble sugar content among the population.

**Conclusions:**

In conclusion, we speculate that the alteration of the SNP in the *NnADAP* can affect the size and sugar content of the lotus rhizome.

**Supplementary Information:**

The online version contains supplementary material available at 10.1186/s12864-021-07473-w.

## Background

Chinese lotus (*Nelumbo nucifera*) is popularly used for food and ornaments with agricultural and economic value. Based on their biological characteristics and observed ecological habits, field visits, and regional experiments, the lotus was documented to exist in two natural ecotypes, namely, temperate lotus and tropical lotus. These two ecotypes have distinctly different growth and development habits, especially in their underground stem development. Temperate lotus has an apparent annual growth and development cycle, withering in autumn while the underground stem expands into lotus rhizome and hibernates. Tropical lotus in a tropical climate will not have an enlarged rhizome but grows into a whip shaped rhizome during the entire growth period [1, 2]. In temperate lotus, the development stages of underground stem could be separated into four (S1 to S4) depending on the morphology [3, 4], S1 is described as the stolon stage, S2 as the middle stage where extension of underground stems occurs, in S3 thickening of underground stems occurs, and in S4 there is a continuous thickening of underground stems as well as simultaneous accumulation of starch and extension of internodes.

The AP2/ERF (APETALA2/Ethylene Responsive Factor) is a sizable transcription factors family in the plant kingdom. Their classification was widely studied in many model plants, including Arabidopsis and rice (*Oryza sativa*) [5], and many non-model plants such as the rubber tree (*Hevea brasiliensis*) [6], tea plant (*Camellia sinensis*) [7], and the Chinese cabbage (*Brassica rapa ssp. pekinensis*) [8]. According to the domain number and gene sequence of the AP2 genes, the massive group has been divided into three subgroups [5], including the AP2 subfamily (containing two AP2/ERFs domain), the ERF subfamily (containing one AP2/ERF domain), and the RAV subfamily with one AP2/ERF domain. Besides, Sakuma classified the AP2/ERF family into five branches, including AP2, DREB, RAV, ERF, and Soloist [9].

The AP2/ERF superfamily members were demonstrated to be involved in plant growth [10–13], response to stress [14–17], and metabolism [18, 19]. The AP2 subfamily genes, *WRINKLED* (*WRI*), are involved in fatty acid production and biosynthesis pathway and have different biological functions in Arabidopsis [20, 21]. Besides, AP2/ERF proteins are involved in the carbohydrate biosynthesis and metabolism-regulation network in storage organs of Arabidopsis [22], rice (*Oryza sativa*) [23], and maize (*Zea mays*) [17, 24]. Nevertheless, previous studies also showed that the AP2 subfamily (L1140) play critical roles in the formation of lotus rhizome [3]. Furthermore, two AP2-like transcription factors (NNU_12870 and NNU_17043) showed the same or an opposite trend with rhizome formation in tropical and temperate lotus [4]. Here we have given an overview of this family and its expression in lotus rhizome, elucidating the relation between the AP2/ERF family and lotus rhizome development.

Quantitative trait loci (QTL) mapping studies provide numerous DNA markers and thus contributing to the improvement of plant breeding via marker-assisted selection (MAS) [25, 26]. The genetic linkage map of various plant types and floral organ characteristics in lotus has accelerated the process of molecular breeding and crop improvement [27, 28]. Although the genetic map with high-density of lotus has been constructed and QTL mapping of traits related to underground stem development has been obtained [29], the verification of specific genes needs to be studied further. Single nucleotide polymorphism (SNP) markers are vital for MAS, as even one SNP in 5′ regulatory region was capable of causing disability of seed shattering in rice [30] and SNP resulting in a truncated SHATTERING 3 (SH3)/SEED SHATTERING 4 (SH4) protein caused the seed shattering disability in *Oryza glaberrima* [31].

Previously, we detected several SNP markers in some genes in lotus, which showed a differential expression pattern between temperate and tropical lotus [4, 32]. To further confirm their potential relationship with the underground stem enlargement phenotypes, we selected one SNP in *NnADAP* (NNU_25830) belonging to the AP2 subfamily to show its preference in lotus varieties with various rhizome morphological characteristics. SNP genotyping showed that this SNP is associated with the agronomic traits of lotus rhizome. Besides, we analysed the expression of *NnADAP* and established a way that linked this marker with lotus rhizome traits.

## Results

### Identification, distribution, and phylogenetic analysis of the AP2/ERF superfamily genes in lotus

The AP2 domain (PF00847) was blasted in the Lotus Database [33], where 121 predicted AP2/ERF proteins were identified. The length of these genes ranged from 278 to 8.01 k bp, and their corresponding polypeptide sequences ranged from 82 to 1.01 k aa (Additional file 1: Table S1). According to sequencing data, except for the soloist gene, the other 120 AP2/ERF family genes were mostly (90.8%) mapped to the ten megascaffolds 1–10 (Additional file 2: Fig. S1A). The rest (9.2%) were distributed in other small megascaffolds (Additional file 2: Fig. S1B). Megascaffold 1 and megascaffold 2 were the largest two megascaffolds (255 and 133 Mb), containing 55% of the total members (Additional file 2: Fig. S1A).

**Fig. 1:**
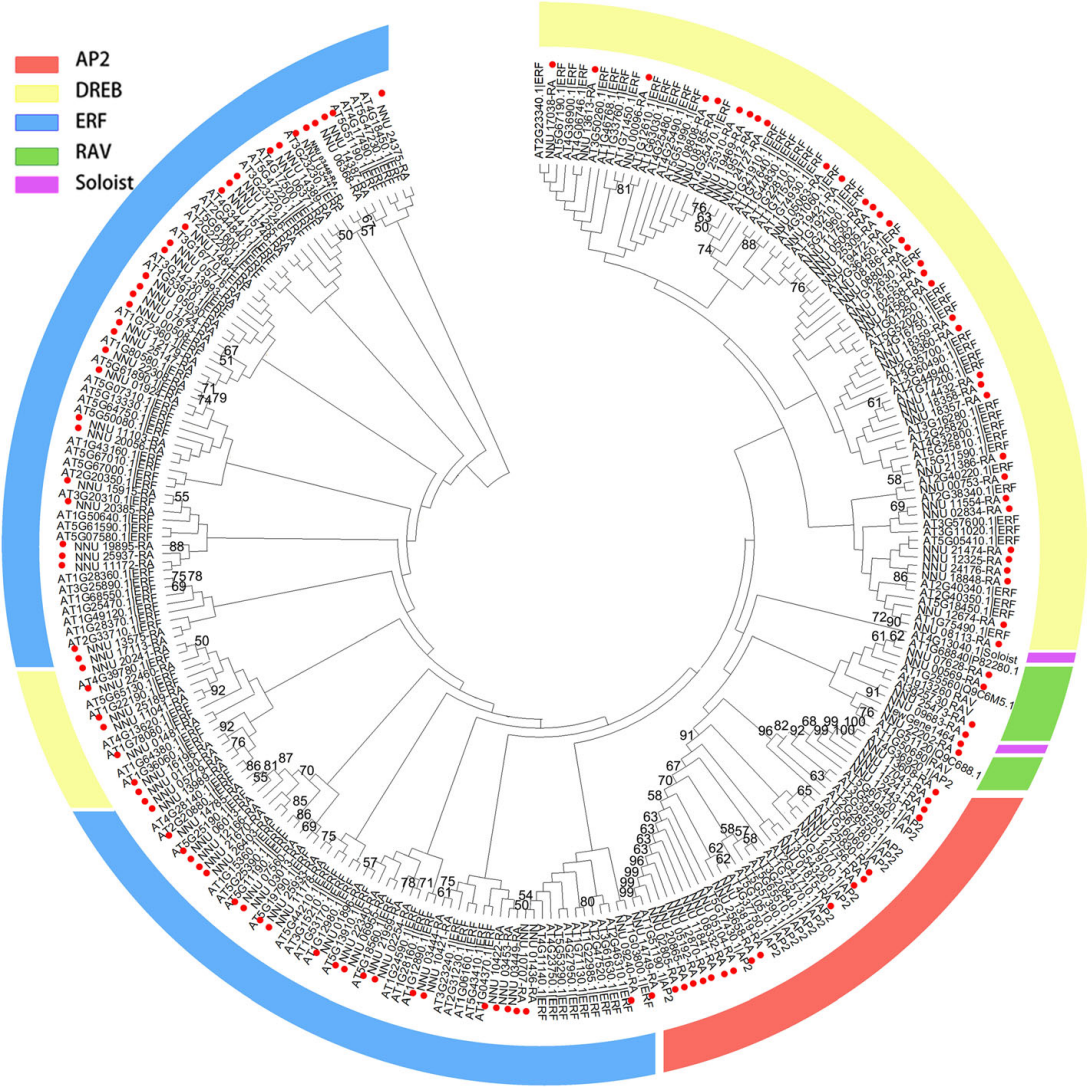
Phylogenetic tree of AP2/ERF genes of *Nelumbo nucifera* and *Arabidopsis.* The evolutionary history was inferred using the Maximum Likelihood method and JTT matrix-based model [35]. This analysis involved 267 amino acid sequences. There were a total of 1215 positions in the final dataset. Evolutionary analyses were conducted in MEGA X [36]. Color blocks of different colors represent different subfamily members. Red pots indicated genes in lotus

We constructed a phylogenetic analysis of AP2/ERF superfamily with *Nelumbo nucifera* and Arabidopsis by employing the maximum likelihood (ML) method. Sequences of 121 NnAP2/ERF genes and 143 AtAP2/ERF genes were retrieved from the Lotus Database [33] and the Arabidopsis information resource website, respectively [34]. The phylogenetic tree in lotus showed that NnAP2/ERF genes were separated into five subfamilies according to the Arabidopsis classification method, including AP2, ERF, DREB, RAV, and Soloist (Fig. 1) [5, 37]. Eighteen genes belonging to the AP2 subfamily, seventeen of which had two AP2 domains and the one gene having only one AP2 domain. There were 55 genes in ERF subfamily and 42 genes belonging to the DREB subfamily, which had only one AP2 domain, and five genes in RAV subfamily with both an AP2 and B3 domain. There was one soloist gene (newGene_1464) sharing a similar protein sequence with Arabidopsis soloist gene At4g13040, which possessed one AP2 domain (Additional file 1: Table S1).

### Structure analysis of lotus AP2/ERF superfamily

**Fig. 2:**
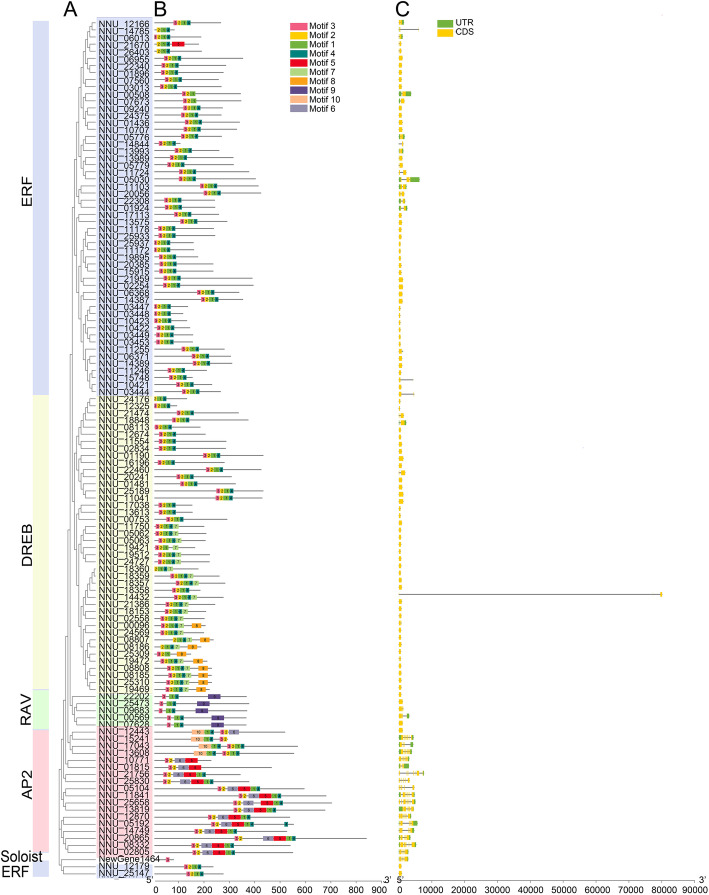
The phylogenetic tree (**a**), motif elicitation (**b**), and gene structure(**c**) of lotus AP2/ERF family. The motifs sequences were listed in Additional file 1: Table S2. Color blocks of different colors represent different subfamily members

After classifying the AP2/ERF superfamily in lotus, we analysed its gene and protein structure. The gene structure analysis was performed through the GSDS (Gene Structure Display Server) 2.0 [38]. The number of exon and intron differed in the four subgroups of the AP2/ERF family. The results showed that genes in AP2 subfamily possess more than ten exons individually. Genes in RAV subcategory and those in the soloist subfamily had two to five exons each, while 29.1% of members of the ERF subgroup had two exons with the remaining genes having only one exon. In the DREB subfamily, most of the genes had one exon with only four genes exceeding one exon (Fig. 2). To further study the characteristics of NnAP2 proteins, the motifs of 121 NnAP2 proteins were analysed using MEME (Multiple EM for Motif Elicitation, V5.0.1 [39]). Ten predicted motifs were set as default parameters and were listed in Additional file 1: Table S2. Members of the AP2 subgroup mainly own four to six motifs. All the AP2 subgroup proteins possessed motif 2 and motif 3 with the majority having motif 1, motif 6, and motif 3. The combination of motif 1, motif 2, motif 3, and motif 4 was similar to those in ERF subfamily. The comparison of DREB AND ERF had 37.8% of DREB subgroup proteins being similar with those of ERF subfamily, while 20% of the proteins had an additional motif (motif 8), and 48.9% had motif 7. All RAV subgroup proteins included motif 3, motif 1, motif 4, and motif 9, while the soloist protein had only motif 3. The structure analyses assembled using TBtools by Chen [40] were shown in Fig. 2. From these results, we deduced that most of the clustered genes in the phylogenetic tree share similar patterns of motif combination and may be involved in similar biological functions.

### Gene expression analysis of the NnAP2/ERFs during lotus rhizome formation

**Fig. 3:**
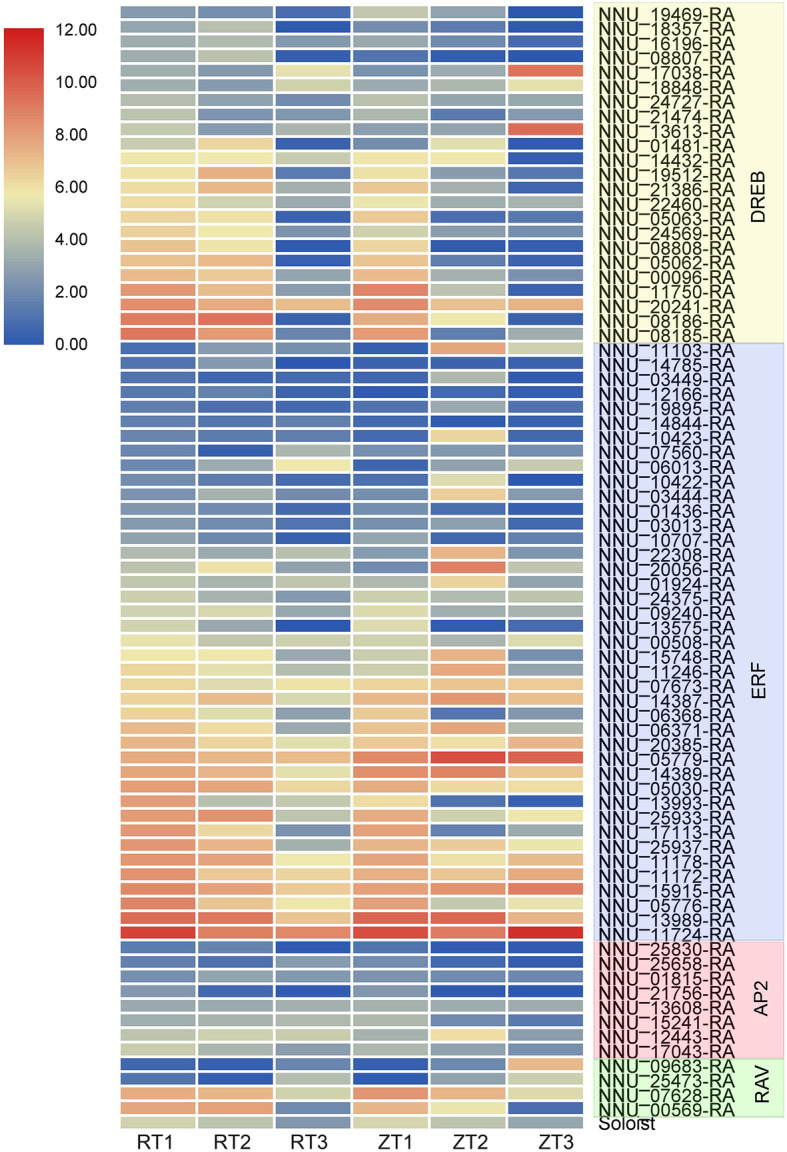
Heat map of the relative expression levels of 77 NnAP2/EFR genes during lotus rhizome formation process of temperate lotus (ZT) and tropical lotus (RT). Color scores were normalized by the log2 transformed counts of RPKM values. Expression differences in the transcripts were clustered by the hierarchical complete linkage clustering method using a Pearson correlation matrix. The heat maps were drawn using the Multi Experiment Viewer (version 4.9.0). Color blocks of different colors represent different subfamily members

To assess the prospective function of NnAP2/ERFs genes in the process of rhizome formation and enlargement, we explored gene expression based on previous RNA-seq data [4]. The results showed that 63.6% of AP2/ERF superfamily members were detected during lotus rhizome formation, including eight AP2 subfamily members, 41 ERF genes, 23 DREB genes, four RAV members, and one soloist gene (Additional file 1: Table S3, Fig. 3). The overall expression of eight AP2 subgroup members was low with NNU_21756 and NNU_17043 showing a decreasing expression along with lotus rhizome enlargement. In contrast, the expression of NNU_12443 reached its peak during the middle stage of rhizome development. The other five genes showed different expressions between temperate and tropical lotus. Four RAV genes and one soloist gene showed no difference in their expression trend during the development process of tropical and temperate lotus underground stem. In DREB subgroup, 82.6 and 91.3% genes were down regulated in the rhizome of temperate lotus and tropical lotus, with a similar trend being observed in the ERF subfamily. More than half of the lotus AP2/ERF superfamily were expressed in lotus rhizome. Most of them showed a different expression pattern between temperate and tropical lotus, which indicates that this family may play a role in rhizome enlargement. We, therefore, focused on the genes that were up-regulated in temperate lotus and down-regulated in tropical lotus.

### Acquisition and verification of target SNP in *NnADAP*

After the second-generation genome sequencing of several lotus varieties with different rhizome perimeters [32], we cloned an AP2 subfamily gene, NNU_25830 (herein known as *NnADAP*), which possesses an interesting SNP (megascaffold_20, 3,578,539) which differ with the change of rhizome perimeter. Then we verified the second-generation genome sequencing results by Sanger sequencing using genomic primers (NnADAP-G forward and reverse primers, Additional file 1: Table S4), and the sanger sequence results mostly kept congruent with high-throughput results (Additional file 1: Table S5). In tropical lotus, the SNP was “TT”, while in temperate lotus, it was “CC” or “C/T”, and the single basement change leads to variation in the amino acid sequence of proline to leucine.

**Fig. 4:**
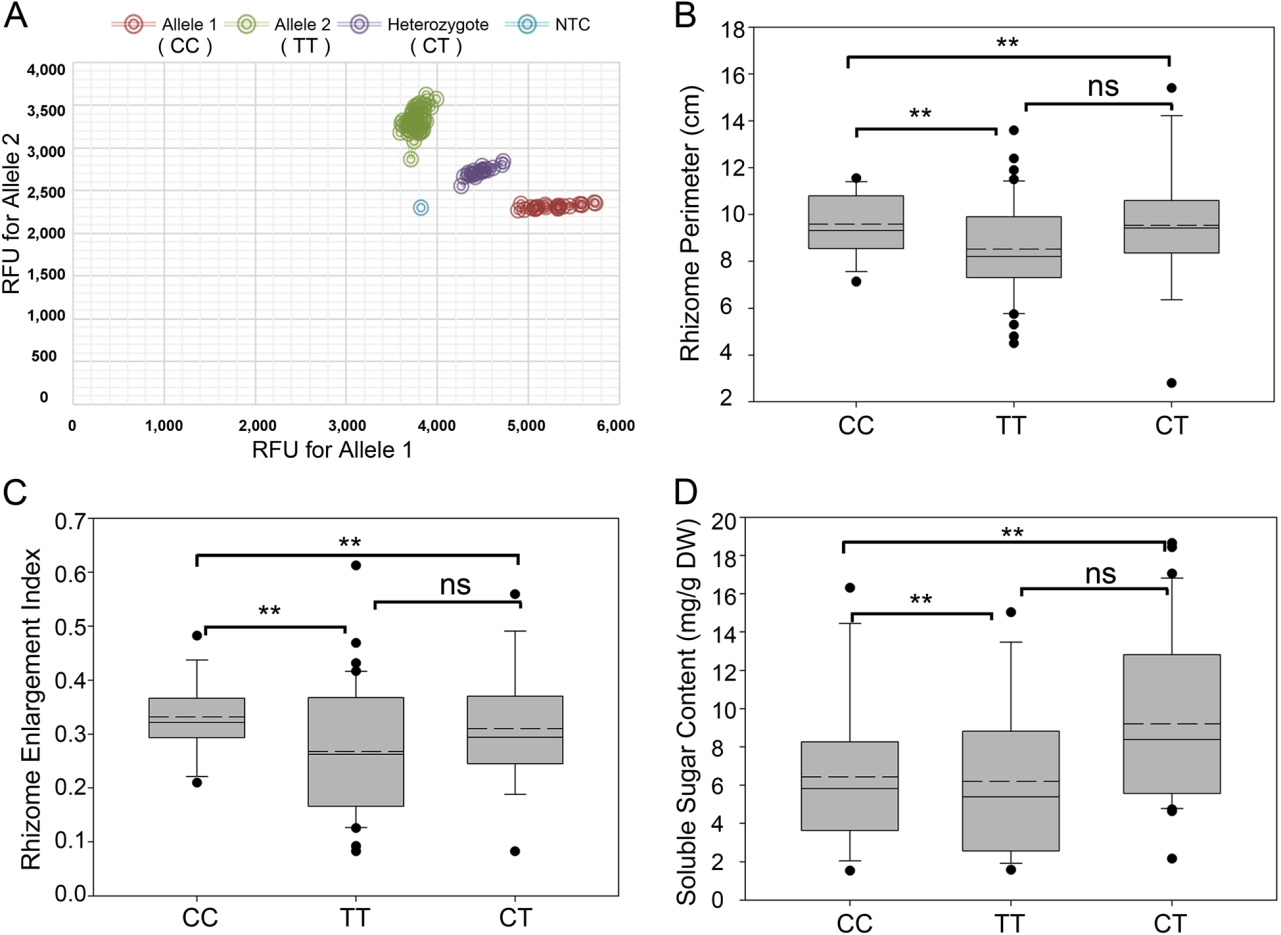
The SNP genotype results and the difference of lotus varieties rhizome traits between three groups with different SNP alterations. **a** SNP genotyping of target single nucleotide. KASP assay showing clustering of lotus varieties on the X-axe (Allele 1 with FAM) and Y-axe (Allele 2 with HEX). RFU is the Relative Fluorescence Units. Red dots represent varieties have the FAM-type allele 1; green dots represent varieties have the HEX-type allele 2; blue dot represents the NTC (non-template control). **b**, **c**, and **d**: Boxplot of Perimeter, REI, and sugar content in group I (SNP genotype: CC), group II (SNP genotype: TT), and group III (SNP genotype: C/T), respectively. The straight line represents median, the dotted line shows mean value and open circles indicate outliers. Upper and lower lines indicate maximum and minimum values. *, *P* < 0.05; **, *P* < 0.01. ns, not significant

To confirm the preference and effect of the SNP in different phenotypes of lotus rhizome, we collected a population of 95 lotus varieties for SNP genotype analysis (Additional file 1: Table S6). Among the population, the genome of 18 varieties have been sequenced [32], and this whole population included wild temperate lotus, cultivated temperate lotus, tropical lotus, *N. lutea*, and several hybrids, depending on the agricultural application and experience in cultivation [32, 41]. We conducted Kompetitive Allele-Specific PCR (KASP) [42] on the flanking sequences containing the target SNP with a pair of KASP primers (KASP forward and reverse primers, Additional file 1: Table S4). After KASP genotyping in the lotus collection, our target SNP had a clear preference in various lotus varieties (Fig. 4a; Additional file 1: Table S6). Thus, we divided the lotus collection into three groups named group I (Allele 1: CC), group II (Allele 2: TT), and group III (Heterozygote: CT) depending on the SNP genotype.

### Association between the target SNP in *NnADAP* and rhizome biological indices in lotus

Lotus rhizome enlargement was accompanied by changes in morphological characteristics and sugar and starch content [43]. In this study, we focused on the character of the three lotus groups to elucidate the function of the target SNP on *NnADAP* in traits of rhizome, including REI, perimeter, and soluble sugar content (Fig. 4b-d, Additional file 1: Table S6). The REI and perimeter in group I were significantly higher than the other two groups. At the same time, there was no difference between group II and group III, implying that lotus rhizome size in the SNP genotype CC plants may be larger. The soluble sugar content in group I showed a significant increase than group II and a significant decrease compared with group III. Group II had the lowest soluble sugar content with the SNP genotype TT, and the other two groups with CC and CT showed a higher sugar content, which probably revealed that SNP CC promotes soluble sugar accumulation.

### The temporal-spatial expression of *NnADAP* in temperate lotus

**Fig. 5:**
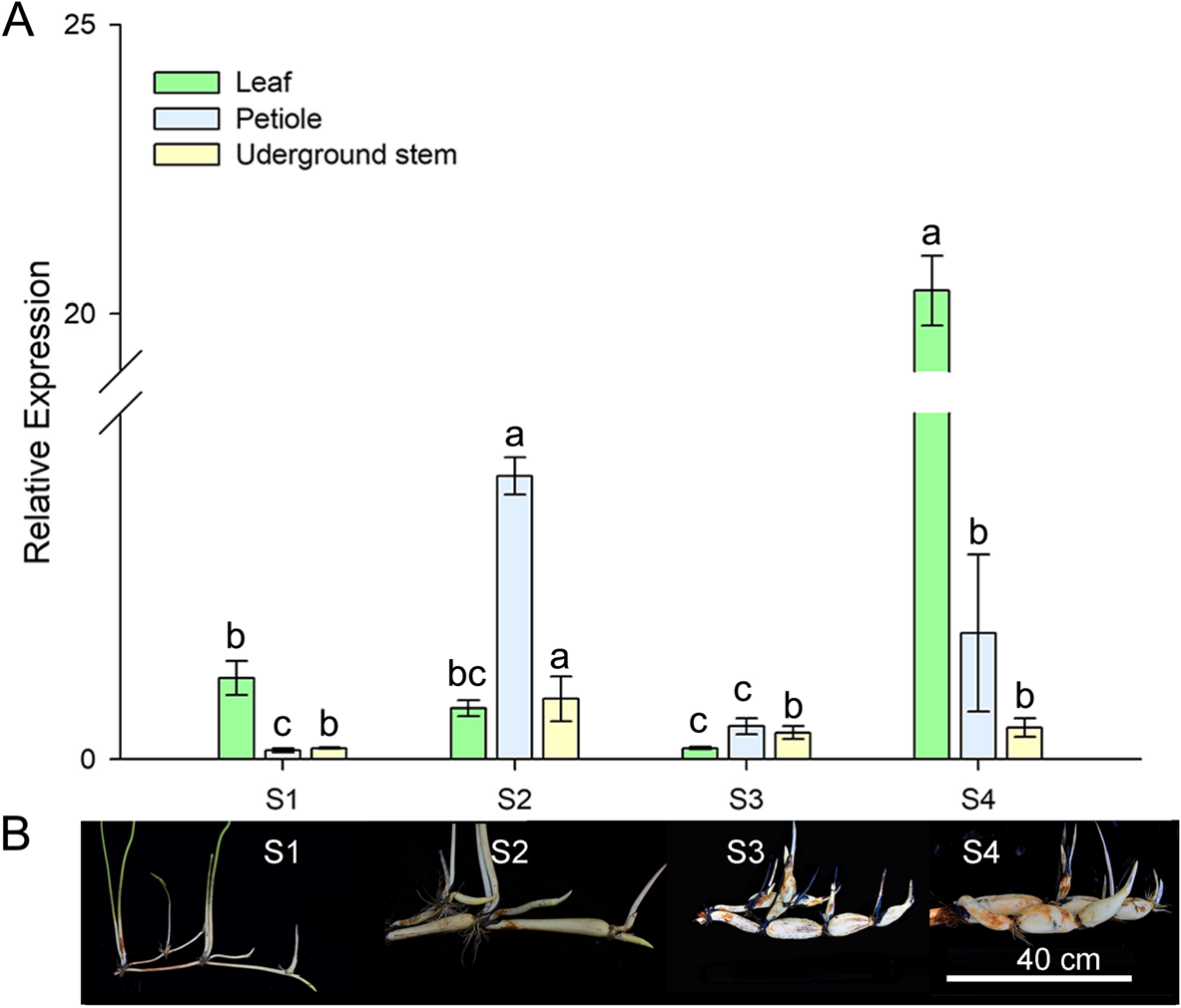
Relative expression level of *NnADAP*. **A** Relative expression level of *NnADAP* during the enlargement process (S1, S2, S3, and S4) of temperate lotus’ underground stem. The green, blue, and yellow bars represent the gene expression at leaf, petiole, and underground stem, respectively. **B** The rhizome of temperate lotus at different development stages

Previous transcriptome data illuminates the differences in *NnADAP* expression in the lotus rhizome development process in temperate and tropical lotus [4]. To further analyse the role played by *NnADAP* (with target SNP CC) in rhizome formation, we investigated the gene expression of critical plant parts during the temperate lotus formation process (Fig. 5). The expression of NnADAP was high in the petiole and rhizome, with the highest expression occurring at S2 stage of rhizome development. In the leaves, the expression of *NnADAP* was exceedingly significantly increased in S4. Together with the increasement of the starch and soluble sugar in temperate lotus rhizome [4, 43], we deduced that *NnADAP* (with target SNP CC) might be involved the accumulation of carbohydrate in leaves and carbohydrate partitioning.

## Discussion

The AP2/ERF family is widespread in plants and has many members that are involved in many phases of plant growth and development, including phytohormones biosynthesis [44, 45], floral organ development [46–48], and stress resistance [49–52]. As the lotus is becoming one of the model plants in the horticultural industry and offering immense contribution and allowing more in-depth studies focused on this species [53, 54], the analysis of lotus AP2/ERF superfamily lotus has a great value. Lotus rhizome has a crispy flavor when fresh and an agreeable taste after simmered in soup. Its richness in water, starch, and vitamin, lotus rhizome was used as a popular vegetable and snack in Asia. Several types of research have focused on food usage of lotus rhizome, including the changes that occur during cooking and some other processing method, including heat blanching [55, 56], the phenols and starches, and ADP-glucose pyrophosphorylase [57–59]. Further studies on lotus gene families and gene markers of economic traits are of great significance to molecular assisted lotus breeding.

### Characteristics of AP2/ERF gene family in lotus

In the present study, we identified 121 NnAP2/ERF superfamily genes in lotus, which consisted of 42 DREB subgroup genes, 55 ERF genes, 18 AP2 members, five RAV subfamily genes, and one soloist gene (Fig. 1). The subfamilies ratio is similar to that in Arabidopsis and longan (*Dimocarpus longan* Lour.) [60], with the total numbers being less than that in Arabidopsis and several other species, including Chinese cabbage(*Brassica rapa ssp. pekinensis*) [8], grape (*Vitis vinifera*) [61], rice [5], and poplar (*Populus trichocarpa*) [62].

According to the gene structure and evolution rates, the AP2/ERF superfamily was separated into five subfamilies [5]. The AP2 subfamily has the most introns and exons, and its protein structure is dominated by the combination of Motif 3, 2, 6, 5, 1, and 4 (Fig. 2). The difference between these members is the number of amino acids between different Motifs. In the DREB and ERF subfamilies, many genes lack introns. The ERF subfamily has 67.3% of genes without intron structure, while in the DREB the number is as high as 83.3%. It also has a similar phenomenon in tartary buckwheat (*Fagopyum Tataricum*) [63] and longan [60]. The protein structures of the ERF subgroup and DREB subgroup has certain similarities. The DREB subfamily protein structure has more Motif 7 and Motif 8 than ERF. The protein structure of the RAV subfamily is the most conserved, with fixed combinations of Motif 3, 1, 4, and 9, while the protein structure of the soloist gene NewGene1464 only lacked Motif 3.

### The expression of AP2/ERF family in lotus rhizome

In this study, eight AP2 subfamily genes expressed in the lotus underground stem with a low-level expression during lotus rhizome formation process, and most of them showed a different expression trend between temperate and tropical lotus. Flowering and tuberization are the representatives of reproductive and vegetative growth, respectively, which share an overlapping plant energy allocation. AP2 and AP2-like genes are involved in the initiation of flower growth [64–67], and some of them cause inhibition of flowering in various flowering plants, including rice, Arabidopsis, and *Arabis alpina* [48, 68, 69]. Flowering and tuber formation were triggered by certain overlapping signals in potato (*Solanum tuberosum*) [70], and the transition between flowering and tuberization was induced by ‘florigen’ and ‘tuberigen’, which share similar signaling pathway [71, 72]. In this study, the gene expression of *NnADAP* reached the highest in lotus rhizome and petiole in the initial of rhizome enlargement at S2 and reached the highest expression in leaf at S4. For the flowering time in temperate lotus is short (several days from S2 to S3), we speculated that *NnADAP* in lotus might also play an antagonistic role between lotus flowering and rhizome development.

DREB and ERF genes are widely researched in various plants focusing on stress resistance. Debbarma et al. [73] reviewed the ERF subfamily members in abiotic stresses tolerance, including drought, salinity, heat, cold, oxidation, and others in many plants, such as Arabidopsis [74], rice [75], tomato (*Lycopersicon esculentum*) [76], and cotton (*Gossypium hirsutum*) [77]. There were 23 genes out of 42 DREB subfamily and 41 genes out of 55 ERF subfamily was expressed in the lotus rhizome (Fig. 3), which might indicate the different roles of AP2/ERF genes in lotus rhizome formation. The expression of DREB and ERF subfamily genes in lotus rhizome was high in the first two stages of lotus rhizome development, which may imply that the gene’s function focuses on responding to environmental stress in the early stage of lotus underground stem development. RAV subfamily members are also involved in stress resistance. Arabidopsis *RAV1* gene adversely regulates drought and salt tolerance and seed development, and gene expression fluctuates because of plant hormones and abiotic stresses [78, 79]. Tomato *SlRAV* along with tomato *SlERFs* enhanced the tolerance to bacterial wilt [80]. In soybean (*Glycine max* L.), RAV genes seem to increase the resistance of salt and drought and might be involved in varying signaling pathways [81]. A total of four RAV subgroup genes and one soloist gene were detected in lotus rhizome and all of them exhibiting the same expression trend with the development of lotus rhizome in tropical lotus and temperate lotus. Among the four RAV genes, two of them showed an increase along with the rhizome formation while the other two genes showed a decreasing expression trend, indicating that lotus RAVs might play opposite roles in rhizome development.

### SNP marker for measuring lotus rhizome production

The SNP marker has been researched in many economic traits of various species, such as maize [82], soybean [83], and longan [60, 84]. With the genetic and molecular studies in lotus, many molecular genetic markers have been researched and developed, including insertions and deletions (InDels), simple sequence repeats (SSRs), and abundant SNPs [32, 85, 86]. In lotus, the genome-wide analysis of SNP on AP2 domain demonstrated that, compared with other transcription factor gene families like MYB and RING finger, AP2/ERF gene family in lotus have a lower gene mutation of a loss of function [32], which implied that AP2 domain suffered a lower selection pressure. The SNPs in the protein-coding region of the AP2 gene are worth further study.

In this study, the target SNP in *NnADAP* showed a significant association between the SNP genotype and some rhizome phenotypes, including the perimeter and length of one rhizome internode, and sugar content, which constitutes the morphological characteristics and main nutritional characteristics of the rhizome. We collected 95 lotus germplasm resources as the SNP genotyping population, including wild temperate lotus, cultivated temperate lotus, and tropical lotus (Additional file 1: Table S6). Most of the flower characters have been collected, and the genome background of some lotus varieties was released [32, 87]. This study focused on the lotus rhizome traits of these lotus germplasm resources. Through the analysis of underground stem morphology data, the REI of this population ranged from 0.08 to 0.61, which achieves our research goal of the biological phenomenon of lotus rhizome enlarging with SNP alter. Perimeter and REI are important morphological indicators of lotus rhizome [88–91]. When the SNP is CC, the group has the maximum perimeter and REI, which shows that CC has a higher contribution to promoting lotus rhizome expansion than CT and TT. Also, the SNP genotyping result of the soluble sugar content of the CC-variety group was consistent with the perimeter and REI trends. This phenomenon reflects that there is a specific correlation between the SNP and the sugar content and the rhizome expansion [92]. After preliminary genotyping and phenotyping, the gene markers are efficient for measuring lotus rhizome production and useful for rhizome lotus breeding.

## Conclusions

In summary, we identified 121 AP2/ERF superfamily genes in lotus, classified them into five subgroups, analysed gene distribution and structure, and enumerated their expression patterns during the development of lotus rhizome. Additionally, we focused on an interesting SNP (C/T) on the *NnADAP* gene in the AP2 subfamily. Through verification and SNP genotyping experiments in the population, it was found that the different genotypes of this SNP correspond to the lotus rhizome phenotypes of the population groups. The lotus rhizome morphology data of group I (SNP: CC) was significantly larger than the other groups, and it had higher soluble sugar content. Together with the expression pattern of *NnADAP*, we concluded that the SNP alteration on this gene influences the phenotype of lotus rhizome.

## Methods

### Plant material

A total of 95 lotus used in this study are planted and preserved in Wuhan Botanical Garden, Chinese academy of sciences, Wuhan, Hubei, China (N30°32′48.33″, E114°25′3.98″). Of the 95 accessions, including 21 Thai lotus, 67 temperate lotus, and seven wild lotus, and all the seeds and plants are from Wuhan Botanical Garden, Chinese academy of sciences (Additional file 1: Table S6). Wuhan Botanical Garden reserves the right of final interpretation. All the lotus germplasms are planted in the Wuhan Botanical Garden and some of them were analysed and sequenced in previous studies [4, 32]. Leaf samples were collected in June in 2017 and then stored at − 80 °C for DNA and RNA extraction. Rhizome samples were collected in April 2018.

### Measurements of rhizome enlargement index, water, and soluble sugar of lotus rhizomes

After we measured the rhizome enlargement index, rhizome samples were dehydrated by using an oven heated at 100 °C for 1 h and kept in a 65 °C-oven to measure constant weight. The soluble sugar content was measured following the anthrone colorimetric method [43].

### Identification and classification of the AP2/ERF genes in *Nelumbo nucifera*

From a previous study conducted by Huang et al. [32], numerous SNPs of lotus genomic variation were obtained followed by selection based on the transcriptome difference between temperate and tropical lotus. Several SNPs were selected including SNP (megascaffold_20:3578539, C/T) in *NnADAP*. *Nelumbo nucifera* genome and protein sequence were analysed from the Lotus database [25, 32, 93] and Arabidopsis AP2/ERF gene sequences were retrieved from the DATF database (http://datf.cbi. pku.edu.cn, last accessed date: April 20, 2016) [38] and TAIR website, (https://www.arabidopsis.org/, last accessed date: April 25, 2016). According to the description of Nakano et al. (2006), the AP2/ERF sequence was determined based on the presence of the AP2 domain, and all putative AP2/ERF proteins were compared with Arabidopsis AP2/ERF proteins to classify them into different groups.

### Acquisition of target SNP and verification of the sequence of NnADAP

We developed specific primers based on the target SNP to verify the transcriptome study of the target SNP of *NnADAP* by employing the sanger sequence. Simply, different varieties’ DNA was extracted as templates to conduct polymerase chain reaction with Primer STAR Max DNA Polymerase (Takara), 10 uM forward and reverse primers each. The PCR products were then ligated on T-vector for sequencing.

### Phylogenetic and conserved motif analysis and gene structure of the AP2/ERF genes

Multiple alignments of Arabidopsis and lotus AP2/ERF protein sequences were carried out using ClustalW with default parameters (Thompson et al., 2002). Unrooted phylogenetic tree of all AP2/ERF proteins was generated with MEGA (V10.0) using the maximum likelihood (ML) method with the following parameters: Poisson correction, pairwise deletion, and 1000 bootstrap replicate [36]. Conserved motifs in *Nelumbo nucifera* AP2/ERF TFs were identified using the motif finding tool MEME (Multiple EM for Motif Elicitation, V5.0.1 [39]) with the following parameters: optimum motif width ≥ 10 and ≤ 200, the maximum motifs numbers 25, and the occurrences of a single motif distributed among the sequences with zero or one per sequence (−mod zoops). The gene structure data was download from LOTUS.DB website (http://lotus-db.wbgcas.cn/) [25, 32, 93] and analysed using TBtools [40].

### RNA isolation and quantitative real-time PCR

The plant samples were collected in different stages of stem development for the qRT-PCR assay (Fig. 5B). Total RNA extraction and cDNA synthesis and qRT-PCR reactions were described as previous study [94]. A pair of primers (NnADAP forward and reverse primers, Additional file 1: Table S4) were designed to investigate the expression profiles of *NnADAP*. The candidate gene was performed in three biological replicates. Relative gene expression was standardized by the expression of lotus β-actin NNU_24864 (Additional file 1: Table S4) and analysed using the 2^-∆∆CT^ method.

### KASP SNP genotyping

The platform of LGC Limited of the KASP (Kompetitive Allele Specific PCR) genotyping technology was employed in the present study. The complete coding sequences of different locus alleles used for KASP assays were generated in lotus dataset [33]. The diagnostics SNP was discovered and KASP primers designed following KASP guidelines carrying FAM and HEX (Additional file 1: Table S4) tails at the 3′ end. The KASP experiment steps were carried out as described in previous study [95]. After initial testing on real-time PCR (BioRad®, CFX-96) system, the *NnADAP* KASP assay was applied to varieties.

## Supplementary Information

**Additional file 1: Table S1.** The AP2/ERF family in *Nelumbo nucfera*. **Table S2.** The motif sequence of lotus AP2/ERF family. **Table S3.** The transcriptome data of AP2/ERF family in the temperate lotus and tropical lotus rhizome. **Table S4.** Primers used in this study. **Table S5**. Detection and the authentication of SNPs in genome sequencing results through first-generation sequencing. **Table S6.** The rhizome traits and SNP phenotype of 95 lotus germplasm resources

**Additional file 2: Figure S1.** A, Mapping of AP2/ERF genes in lotus main megascaffolds. The unit of the length of each megascaffold is megabase. B, The numbers of these genes in each megascaffold

## Data Availability

*Nelumbo nucifera* genome and protein sequence analysed during the current study are downloaded from the Lotus database [25, 32, 93] and Arabidopsis AP2/ERF gene sequences are available in the DATF database (http://datf.cbi. pku.edu.cn) [38] and TAIR website (https://www.arabidopsis.org/). The information of lotus genome was downloaded from the NCBI Sequence Read Archive SRA with the accession number SRP021228 (http://trace.ncbi.nlm.nih.gov/Traces/sra/?study=SRP021228) and the transcriptome dataset analysed during the current study are available in NCBI SRA (http://www.ncbi.nlm.nih.gov/Traces/sra) with accession number SRA271278 from previous studies [4, 33].

## Abbreviations

AP2/ERF: APETALA2/Ethylene Responsive Factor
QTL: Quantitative trait loci
MAS: Marker-assisted selection
SNP: Single nucleotide polymorphism
ML: Maximum likelihood
GSDS: Gene Structure Display Server
MEME: Multiple EM for Motif Elicitation
KASP: Kompetitive Allele-Specific PCR
RFU: Relative Fluorescence Units
InDels: Insertions and deletions
SSR: Simple sequence repeat
