# Supplementary material for: Genome-wide analysis of AP2/ERF superfamily in lotus (Nelumbo nucifera) and the association between NnADAP and rhizome morphology
Source: BMC Genomics. 2021 Mar 9;22:171. doi: 10.1186/s12864-021-07473-w (PMC7945336; doi:10.1186/s12864-021-07473-w)
Supplement: Supplementary file 2 — Additional file 2: Figure S1. A, Mapping of AP2/ERF genes in lotus main megascaffolds. The unit of the length of each megascaffold is megabase. B, The numbers of these genes in each megascaffold [file 12864_2021_7473_MOESM2_ESM.docx]

**Additional file 2.**


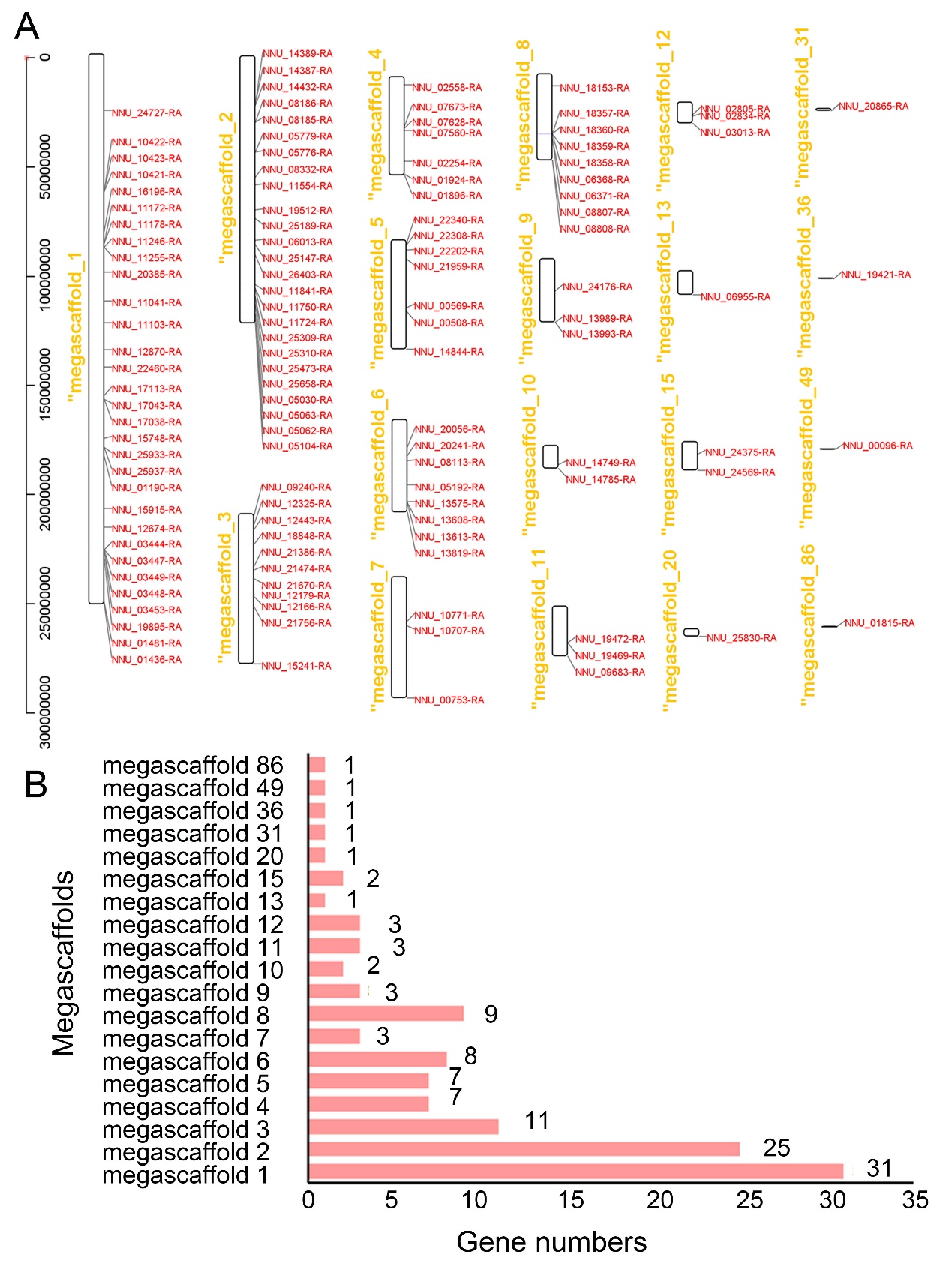


Figure S1. A, Mapping of AP2/ERF genes in lotus main megacaffolds. The unit of the length of each megascaffold is megabase. B, The numbers of these genes in each megascaffold.
